# Associations between body mass index, mid-arm circumference, calf circumference, and functional ability over time in an elderly Taiwanese population

**DOI:** 10.1371/journal.pone.0175062

**Published:** 2017-04-11

**Authors:** Hsin-Jen Tsai, Fu-Kuei Chang

**Affiliations:** Department of Health Management, I-Shou University, Kaohsiung, Taiwan; Istituto Di Ricerche Farmacologiche Mario Negri, ITALY

## Abstract

**Background:**

Anthropometric measurements such as body mass index (BMI), mid-arm circumference (MAC), and calf-circumference (CC) are assessed with ease during regular health visits, but the associations between these anthropometric parameters and functional ability in elderly population over time has not been studied in detail. This study aimed to examine the associations between functional ability and the anthropometric parameters BMI, MAC, and CC in Taiwanese adults ≥ 65 years old.

**Methods:**

Data were obtained from the Taiwan Longitudinal Study of Aging and analyzed retrospectively.

**Results:**

Functional decline over a 4- and 8-year period was noted in approximately 14% and 21% of study participants, respectively. BMI was negatively associated with participants’ current Activities of Daily Living (ADL) scores, and was positively associated with 4-year ADL scores in adults ≥ 65 years old (β = -1.19 and 1.14, P = 0.0010 and 0.0420, respectively). MAC and CC were negatively associated with current ADL scores (β = -1.46, P < 0.0001 and β = -4.68, P < 0.0001, respectively). The association between CC and current ADL score was stronger than the association between current ADL score and either BMI or MAC. For adults ≥ 65 years old, a high BMI increased the risk of ADL decline over 4 and 8 years by 4-fold and 3-fold (adjusted odds ratio = 4.23 and 2.64, 95% confidential interval = 1.95–9.19 and 1.22–5.71, P = 0.0003 and 0.0141, respectively).

**Conclusions:**

BMI is a significant predictor of decline of functional ability in Taiwanese adults ≥ 65 years old. CC is an important anthropometric indicator of current functional ability among older adults.

## Introduction

Taiwan is one of the most rapidly aging societies in the world. The proportion of adults ≥ 65 years old was 11% in 2010, and is expected to reach 20% in 2025 [1, 2]. As a consequence, aging and aged-related issues have become a public health concern. In 2013, the World Health Organization highlighted the importance of frailty in an aging society [3]. Frailty in the elderly is related to aging, functional disability and comorbidity [4]. One of the major phenotypes of frailty in older adults is a loss of their ability to independently perform activities of daily living (ADLs) [5, 6]. It is estimated that close to 15% of elderly adults ≥ 70 years old experience some limitation in their ability to perform ADLs [7], and this increases to up to 46% in institutionalized adults ≥ 65 years old [8]. This decline in functional ability negatively impacts the health and quality of life of older adults, and ultimately leads to a higher mortality rate and a heavy societal burden [9–11]. Therefore, it is of great importance to society and its members to identify factors which can predict this decline in functional ability, and to delay or prevent its occurrence altogether.

Weight, height, and body mass index (BMI) are parameters which are easily obtained as part of patients’ regular physical checkups, and are frequently used anthropometric tools. Previous studies have reported that these factors are associated with health status, disability, and mortality in the elderly [12–15]. Other simple anthropometric parameters include mid-arm circumference (MAC) and calf circumference (CC) [13], and these were reported to be associated with mortality in the elderly institutionalized population [8]. However, MAC and CC are less frequently used anthropometric measurements, and the associations between these and functional ability have received little attention.

The proportions of frail and pre-frail adults ≥ 65 years old in Taiwan is approximately 4.9% and 40%, respectively [5]. Delaying the onset of frailty can improve the quality of life and decrease the mortality of older adults. Early detection of frailty syndrome in the elderly is becoming a priority among medical practitioners, as is identifying predictors of frailty which may help to anticipate and ideally prevent a decline in functional ability. Functional ability in old adults is often defined as the ability to carry out ADL, including bathing, dressing, and other independent living skills, such as shopping and housework. ADL functions are concerned with an old adult’s self-care, whereas independent living functions are concerned with self-reliant functioning in a given environment. The evaluation of functional ability in old adults in the present study is focused on the ADL evaluation. Anthropometric measurements such as BMI, MAC, and CC, are simple, non-invasive and easy-to-use tools in routine health assessments. However, the associations between these and functional ability over time in older adults has not been investigated in detail, and data on the ability of these parameters to predict changes in functional ability over time are limited. Therefore, our hypothesis is that the anthropometric measurements, BMI, MAC and CC, were associated with subsequent ADL. The present study examined the cross-sectional and longitudinal associations between BMI, MAC, CC and functional ability and its decline in Taiwanese adults ≥ 65 years old.

## Methods

### Study design

This study is based on data from the Taiwan Longitudinal Study of Aging (TLSA) which was provided and managed by the Health and Welfare Data Science Center (HWDC), Ministry of Health and Welfare, Taiwan. As a population-based longitudinal study with a national representative sample launched in 1989, TLSA evaluates the health and well-being of nearly-old and old individuals in Taiwan. Details of the study design and method have been described and documented elsewhere [16]. TLSA uses a stratified multi-staged equal probability sampling design with township as the primary sampling unit. First, TLSA recruited 4049 nearly-old and old adults ≥ 60 years old in 1989 to undertake a survey, and regularly followed up these participants every 3 to 4 years [17, 18]. Participants aged with time and thus, with the same sampling and design, TLSA recruited 2462 and 1599 new participants aged 50–66 years old in 1996 and 2003, respectively, in order to replenish the proportion of young-old population. In total, six waves of TLSA were completed (1989, 1993, 1996, 1999, 2003 and 2007). TLSA excluded Taiwanese aboriginals.

An in-home and in-person questionnaire was conducted by trained investigators. Questions included socio-demographic, lifestyle, health, social functioning, and healthcare information. The 1999 survey uniquely collected nutritional and dietary data, including some anthropometric parameters. Thus, data regarding the anthropometric parameters MAC and CC were only available in the 1999 survey. This study selected the 1999 survey data as a baseline dataset and the 2007 survey as the end-point. In total, 4440 participants aged ≥ 53 years old completed the survey in 1999. Of those, 3778 and 3132 participants were successfully traced and interviewed in the 2003 and 2007 surveys, respectively. TLSA was approved by government-appointed representatives in Taiwan. All study participants provided informed written consent. All identified information of participants were removed when data were released from the HWDC, Ministry of Health and Welfare, Taiwan. Therefore, the data was analyzed anonymously. The present study was a secondary analysis of TLSA, was reviewed and review exemptions had been approved by Institutional Review Board of the E-Da Hospital.

### Anthropometric parameters

Anthropometric parameters included height, weight, MAC and CC. Self-reported height and weight were used to calculate BMI. BMI (kg/m^2^) was calculated as weight in kilograms divided by height in square meters. MAC and CC were measured by trained investigators. Anthropometric parameters have population- and gender-specific characteristics [19]. In order to avoid the effects of population and sex, measured values of anthropometric variables were standardized for analysis. BMI, MAC and CC were individually divided by values that were Taiwanese-specific anthropometric points, and which were used in the Taiwan version of the Mini-Nutritional Assessment for the elderly [20, 21]. This was done by dividing each participant’s (male and female) BMI by 21 (kg/m^2^). MAC was divided by 23.5 (cm) for men and 22 (cm) for women, while CC was divided by 30 (cm) for men and 27 (cm) for women. Standardized BMI, MAC and CC values were continuous variables and were used in the linear and logistic regression analyses.

### Functional ability

We evaluated functional ability in our study population using a six-item basic ADL assessment. Evaluation of ADLs included the need for assistance while bathing, dressing/undressing, eating, getting out of bed/standing up/sitting in a chair, moving around the house, and going to the toilet [22]. Each item was scored from 0 (no difficulty) to 3 (unable to do it). We then calculated the change in ADL score over 4 and 8 years.

### Other variables

Other variables included socio-demographic factors, lifestyle factors, and co-morbid conditions. Socio-demographic variables included gender, age and years of formal education. Lifestyle factors included smoking, alcohol consumption, and exercise. Smoking habits were ascertained from the following question: “do you smoke and “how many cigarettes do you smoke per day?” Responses were “yes/no” and “none, 1–4, 5–9, and ≥ 10 cigarette/day”, respectively. Alcohol consumption was ascertained by asking the question “Do you drink alcohol?”, and responses were recorded as “yes/no”. Exercise habits were ascertained by asking: “Do you usually exercise?” and responses were “no, ≤ 2, 3–5 and ≥ 6 times/week (wk)”. Response categories were regrouped as “≤ 2 times/wk” and “≥ 3 times/wk”.

Co-morbid conditions were assessed by counting the number of self-reported medical conditions, including hypertension, diabetes, heart diseases, stroke, cancer, lung diseases, arthritis/rheumatism, gastric ulcer/gastric diseases, liver/gallbladder diseases, hip fracture, cataract, kidney disease, gout and bone spurs.

### Data analysis

Descriptive data were weighted for the sampling design. Multivariate linear regression analyses were performed to evaluate the cross-sectional and longitudinal associations between BMI, MAC, CC and current and subsequent ADL scores after adjusting for confounding factors. Confounding factors included socio-demographics, lifestyle factors, and the number of comorbid diseases. Multivariate logistic regression analyses were performed to evaluate the longitudinal associations between BMI, MAC, CC, and functional decline over a 4 and 8 year period. Study subjects were elderly adults ≥ 65 years old. Functional decline was defined as a reduction in ADL score ≥ 1 points over 4 and 8 years. BMI, MAC and CC were incorporated into linear and logistic regression models individually to avoid collinear effects. The correlation coefficients between BMI and MAC, MAC and CC, and BMI and CC were 0.57, 0.55, and 0.59, all *p* < 0.0001. Since CC could be affected by the presence of leg edema, the data obtained from subjects with edematous legs were excluded from the analysis. The β coefficients, adjusted odds ratio (aOR) and 95% confidential intervals (95% CI) were reported. The SAS statistical software package (SAS Institute Inc, Cary, NC, USA) version 9.1 was used for all statistical analyses. Statistical significance was evaluated as alpha = 0.05. Probability levels of P < 0.01 and P < 0.001 were also given.

## Results

Table 1 shows the baseline characteristics of the study participants. The average age of the study population was 73 years old, and participants had an average of 2 comorbidities. The average ADL score at baseline was 0.93. The average BMI, MAC and CC were 23.17 kg/m^2^, 27.96 cm, and 33.35 cm, respectively. When ADLs were reassessed at 4 and 8 years after baseline, the proportion of adults who experienced functional decline was 13.70% and 20.76%, respectively.

**Table 1:** Characteristic of study participants at baseline^a^.

	N = 2988
Age (y)	73.26 ± 5.73
Education (y)	4.85 ± 3.91
Smoking (%)	23.33
Number of cigarette per day (%)	
0	86.36
1–4	1.32
5–9	2.17
≥ 10	10.15
Drinking (%)	22.92
Exercising (%)	55.14
Frequency of exercise per week (times/week) (%)	
0	38.94
≤ 2	5.92
3–5	9.98
≥ 6	45.15
Number of diseases	2.16 ± 1.58
ADL score	0.93 ± 2.98
BMI (kg/m^2^)	23.17 ± 3.07
MAC (cm)	27.96 ± 3.46
CC (cm)	33.35 ± 3.41
Functional decline (%)^b^	
4-year	13.70
8-year	20.76

**Abbreviation:** ADL, Activities of Daily Living; BMI, Body Mass Index; CC, Calf Circumference; MAC, Mid-Arm Circumference.

^a^ Values were weighting-adjusted according to sampling design.

^b^ Functional decline was defined as a reduction in ADL score ≥ 1 points.

Table 2 shows the multivariate linear regression analysis of the associations between standardized baseline anthropometric measurements (BMI, MAC, CC) and current and subsequent ADL scores. After adjusting for confounding factors, the standardized baseline BMI was negatively associated with the baseline ADL score (β = -1.19, P = 0.0010), and positively associated with the ADL score 4 years after baseline (β = 1.14, P = 0.0420). The standardized baseline MAC was negatively associated with the baseline ADL score (β = -1.46, P < 0.0001), as was the standardized baseline CC (β = -4.68, P < 0.0001). None of standardized baseline anthropometric measurements were associated with the ADL score obtained 8 years after baseline.

**Table 2:** Multivariate linear regression analysis of the associations^a^ of standardized baseline BMI, MAC and CC with current and subsequent ADL scores.

	ADL score								
	At baseline			At 2003			At 2007		
	N	β	P	N	β	P	N	β	P
Std BMI	2818	-1.19	0.0010	2137	1.14	0.0420	1635	1.13	0.1579
Std MAC	2859	-1.46	<0.0001	2177	0.34	0.5131	1655	-0.98	0.2042
Std CC	2839	-4.68	<0.0001	2162	-1.39	0.0529	1642	-1.00	0.3422

**Abbreviation:** ADL, Activities of Daily Living; BMI, Body Mass Index; CC, Calf Circumference; MAC, Mid-Arm Circumference; Std, Standardized.

^a^ Adjusted for gender, age, years of formal education, lifestyle factors and the number of diseases.

Table 3 shows the multivariate logistic regression analysis of the longitudinal associations between the standardized baseline values for BMI, MAC, and CC, and subsequent ADL decline over 4 and 8 years. The standardized baseline BMI was positively associated with ADL decline over 4 and 8 years (aOR (95% CI) = 4.23 (1.95–9.19) and 2.64 (1.22–5.71), P = 0.0003 and 0.0141, respectively), while standardized MAC and CC were not.

**Table 3:** Multivariate logistic regression analysis of the longitudinal associations^a^ of standardized baseline BMI, MAC and CC with subsequent ADL decline^b^ over 4 and 8 years.

	ADL decline 1999–2003			ADL decline 1999–2007		
	N	OR (95%CI)	P	N	OR (95%CI)	P
Std BMI	2137	4.23 (1.95–9.19)	0.0003	1635	2.64 (1.22–5.71)	0.0141
Std MAC	2177	1.48 (0.74–2.97)	0.2706	1655	1.06 (0.50–2.25)	0.8734
Std CC	2162	1.13 (0.43–3.01)	0.8063	1642	1.22 (0.44–3.43)	0.7038

**Abbreviation:** ADL, Activities of Daily Living; BMI, Body Mass Index; CC, Calf Circumference; MAC, Mid-Arm Circumference; Std, Standardized.

^a^ Adjusted for gender, age, years of formal education, lifestyle factors and the number of diseases.

^b^ Functional decline was defined as a reduction in ADL ≥ 1 points.

## Discussion

This study demonstrated that BMI, MAC and CC correlate with current and subsequent ADL scores. Moreover, the correlations between these variables and functional ability and decline at 4 and 8 years are independent of each other.

### Body mass index

Cross-sectional results indicated that participants with a high current BMI have a good baseline functional ability, but a high risk of subsequently impaired functional ability. Moreover, in adults ≥ 65 years old, a high baseline BMI significantly increases the risk of ADL decline over 4 and 8 years by 4-fold and 3-fold, respectively. Longitudinal aging studies in the United States and the United Kingdom have indicated that a high past BMI was associated with an increased risk of subsequent functional disability [14, 15]. A high BMI increases the risk of chronic diseases, increases the wear and tear on joints, reduces exercise flexibility, and ultimately causes functional limitations and disability [23]. Our results are consistent with those of previous studies, and suggest that BMI can predict functional decline 4 and 8 years later in adults ≥ 65 years old.

### Calf circumference

Analysis results indicated that CC was associated with current functional ability, but not with subsequent ADL score and ADL decline at 4 and 8 years after baseline. A high CC reflects a better current functional ability in elderly Taiwanese adults. In a study of elderly individuals living in long-term care facilities, CC was found to be significantly correlated with physical function [24]. A higher CC was also associated with a better functional performance in community-dwelling elderly adults [25, 26]. In our study, the β coefficient for the association between CC and current ADL scores (β = -4.68) was higher by nearly 4-fold and 3-fold than those for BMI (β = -1.19) and MAC (β = -1.46). Therefore, CC (rather than BMI and MAC) more accurately reflects the current functional ability of elderly adults. A higher CC indicates more skeletal muscle mass and strength [25, 26], and thus it is not surprising that elderly adults with a higher CC consequently have a better current functional performance. Therefore, CC is a significant predictor of the current functional ability of old adults.

### Mid-arm circumference

This study observed that a baseline MAC was negatively associated with current ADL score in Taiwanese adults ≥ 65 years old. In the Nagoya Longitudinal Study of Frail Elderly, MAC was significantly correlated with the ADL score [27]. In this study, we found that MAC was not associated with subsequent ADL score or with ADL decline over 4 and 8 years. Thus, MAC is not an accurate predictive factor of subsequent ADL score and functional decline. These results are consistent with those of a previous study of community-dwelling men and women >70 years old, which concluded that arm circumference was not associated with subsequent ADL impairment [12]. Furthermore, a study of Japanese community-dwelling frail elders observed that MAC was not an independent predictor of ADL decline during a 2-year period [28]. The ability to independently perform ADLs depends mostly on the presence of adequate mobility, and requires minimum mass and strength in the upper and lower limbs. Taken together, the results described by previous studies and our own suggest that MAC correlates poorly with functional ability as defined by the ability to perform ADLs. Furthermore, MAC was not associated with subsequent functional ability or decline in functional ability.

Observations for the associations between the anthropometric parameters and instrumental ADL (IADL) are limited. These observations focused on the associations of BMI with IADL and reported that BMI were associated with IADL scores [29–33]. BMI was significantly and positively correlated with IADL scores [29]. A longitudinal study reported that BMI values ranging between 23 and 27 was significantly associated with lower risks of IADL functional declines in the subsequent 5 years in healthy France elders [30]. Old adults with low and high BMI had increased risks of developing IADL disability [31–33].

Previous studies investigated the correlations between the anthropometric parameters and cognition and comorbidities. Some studies described the associations of BMI with cognition and reported that BMI was positively associated with risks of cognitive decline [30, 34–36]. A longitudinal study observed that BMI values ranging between 23 and 27 was associated with lower risks of cognitive declines in the subsequent 5 years for healthy community-dwelling old adults [30]. A high baseline BMI was associated with an increased follow-up risk of cognitive decline [34]. Population-based studies indicated that increased BMI in middle age was a risk factor for dementia at later life [35, 36]. Moreover, the correlations of the anthropometric measurements with comorbidities were observed, especially for BMI. A cohort study reported that increasing BMI categories and obesity were significantly associated with multiple morbidity in UK adults aged ≥ 30 years [37]. In a 10-year prospective study, BMI was reported as a predisposing risk factors for multiple morbidities among initially disease-free population [38].

### Strengths and limitations

This study has a number of strengths, including the fact that it is a prospective population-based study which recruited a large number of Taiwanese participants aged ≥ 65 years old. Furthermore, the anthropometric parameters MAC and CC were obtained by trained investigators rather than relying on self-reported measurements. The analysis model was tested at different time points and served as a predictor of change in function ability over time. Predictive analyses were performed for the anthropometric variables BMI, MAC, and CC. Nevertheless, despite its contributions, this study does have certain limitations. First, BMI was calculated using self-reported height and weight, and given that elderly individuals are more likely to report their adult height, which is likely higher than their current height, it is possible that there was some inaccuracy in these measurements. Second, height and weight data may be inaccurate for those participants who had not recently visited a health provider or been weighed, and as a consequence, BMI may have been underestimated [30]. Although we obtained baseline data of the anthropometric measurements of interest, we did not consider any changes which may have occurred in these variables throughout the follow-up period. This is partly due to the fact that MAC and CC were not measured regularly in the TLSA, and thus this information was unavailable in the follow-up surveys. Therefore, we were unable to monitor changes in MAC and CC, and thus unable observe the relationship between these variables and functional decline. Third, more sophisticated anthropometric measurements such as dual energy X-ray absorptiometry (DEXA), were unavailable in the TLSA. Fourth, for old adults, functional ability has a broad range. Functional ability may include abilities for activities of daily living and independent living skills. However, in the present study, we focused on the associations of BMI, MAC and CC with ADL. Independent living ability for old adults were usually evaluated by IADL. IADL was not included in this analysis. The further investigations of the associations of BMI, MAC, and CC with IADL may be needed. Fifth, measurements of functional status were self-reported and the study lacked performance-based measures of functional ability. In TLSA, ADL was evaluated using self-reported information. Although a source of bias needs to be considered, studies indicated that self-reported data on functional disability have adequate validity and were consistent with medical diagnoses and physical tests [39]. Sixth, recall bias and misclassifications cannot be ruled out. Data missing may be due to participants’ cognition, health status, and frailty. Frailty old adults or those with cognitive impairments may be excluded from our analysis due to incomplete ADL evaluations and anthropometric measurements. Thus, selection bias might exist. The findings described above may not be generalizable to other populations with different races, cultures and lifestyles.

### Implication

Our findings have relevant implications for clinicians and public health organizers. These finding supports the routine measurements of BMI, MAC and CC in old persons in the clinical setting would be useful. BMI, MAC and CC measurements offers several advantages. It is easy to perform, less time consuming and not limited by functional capacity, cognition and comorbidities. Anthropometric assessments are efficient tools as the first step in screening old adults to identify those at increased risk of functional decline and disability and to target disability prevention interventions. We increase the awareness of the clinical relevance of these easy-used and easy-measured anthropometric measures on the functional ability. Early detection of clinical functional decline and disability can be achieved. Early detection in turn may help to lessen future disability and it increases the number of disability-free years among old people.

## Conclusions

This study demonstrated that BMI, MAC, and CC are all associated with current functional ability for elderly adults ≥ 65 years of age. A high BMI significantly increases the risk of subsequent ADL decline over 4 and 8 years. Compared to BMI and MAC, a high CC is a significant predictor of current functional ability in adults ≥ 65 years old. Finally, MAC was not related with either subsequent functional ability or decline of functional ability in this study population. The results of this study suggest that BMI can be used as a long-term predictor of functional decline in elderly Taiwanese adults ≥ 65 years old, while CC is a good indicator of current functional ability.
